# Atypical presentation of invasive meningococcal disease caused by serogroup W meningococci

**DOI:** 10.1017/S0950268819002152

**Published:** 2020-01-27

**Authors:** C. Stinson, C. Burman, J. Presa, M. Abalos

**Affiliations:** 1 Medical Development & Scientific and Clinical Affairs, Pfizer Inc, 500 Arcola Rd, Collegeville, PA 19426, USA; 2 Medical and Scientific Affairs, Pfizer Inc, 500 Arcola Road, Collegeville, PA 19426, USA; 3 Medical Affairs, Pfizer SRL, Colectora Panamericana 1804, 1° piso, Sector “B” lado Sur, B1607EEV, Villa Adelina, Buenos Aires, Argentina

**Keywords:** Acute gastrointestinal symptoms, invasive meningococcal disease, pneumonia, septic arthritis, serogroup W

## Abstract

*Neisseria meningitidis*, a gram-negative diplococcus, is typically an asymptomatic coloniser of the oropharynx and nasopharynx. Passage of *N. meningitidis* into the bloodstream can cause invasive meningococcal disease (IMD), a potentially life-threatening illness with rapid onset that generally presents as meningitis, septicemia or both. Serogroup W IMD has been increasing in prevalence in recent years, and observations suggest that it may present with atypical signs and symptoms. Herein, a literature search was performed to identify trends in atypical serogroup W IMD presentation in order to review those that are most prevalent. Findings indicate that the most prevalent atypical presentations of serogroup W IMD include acute gastrointestinal (GI) symptoms, septic arthritis and bacteremic pneumonia or severe upper respiratory tract infection, notably epiglottitis. Atypical clinical presentation is associated with higher case fatality rates and can lead to misdiagnoses. Such risks highlight the need for clinicians to consider IMD in their differential diagnoses of patients with acute GI symptoms, septic arthritis or bacteremic pneumonia, primarily in regions where serogroup W is prevalent.

## Introduction

*Neisseria meningitidis* is a gram-negative diplococcus that asymptomatically colonises the oropharynx and nasopharynx in ~10% of humans [1, 2], providing a reservoir for transmission. Although the bacteria are typically asymptomatic colonisers, bloodstream invasion can occur, resulting in invasive meningococcal disease (IMD). The mechanisms that lead from colonisation to invasive disease are still not entirely understood but are largely attributed to host susceptibility, environmental conditions and meningococcal virulence factors [1, 2]. Polysaccharide capsule expression protects *N. meningitidis* from opsonisation and phagocytosis by host immune cells [1].

IMD has a rapid onset [3] and causes severe illness often associated with high mortality and morbidity, including long-term sequelae (e.g. amputations, hearing loss and neurodevelopment deficiencies) [1, 2]. Efficient clinical recognition of IMD, which often relies on principal signs and symptoms for diagnosis, and initiation of appropriate medical treatment are imperative in improving a patient's chances of a favourable outcome. Disease most frequently presents as either meningitis, septicemia or a combination of both. Classic symptoms of meningitis include fever, intense headache, stiff neck, vomiting or changes in consciousness. Purpura or petechial rash, asthenia or arterial hypotension are classic signs of meningococcemia [2]. Other less common forms of IMD include septic arthritis, pericarditis, gastroenteritis and invasive pneumonia, and present with symptoms different from those seen with meningitis or septicemia [3].

Meningococci are divided into serogroups by the type of polysaccharide capsule they produce [2]. Serogroups A, B, C, W, X and Y cause the majority of disease burden [1]. The sequencing of similar housekeeping genes that are relatively well conserved allows for grouping into sequence types (ST; e.g. ST-11, ST-32) using the multilocus sequence typing (MLST) technique. A clonal complex (cc; e.g. cc11) is a group of similar STs and is named after the most genetically typical and persistent ST in the group. A single ST can belong to multiple serogroups (e.g. ST-11 can belong to serogroup B, C or W) [4].

### Timeline of serogroup W meningococci emergence

Serogroup W meningococci (MenW), sometimes referred to as serogroup W-135 meningococci, were first identified in the 1960s [4]. Throughout the 1990s, MenW was responsible for only 2.6% to 4% of IMD cases in the United Kingdom (UK), France and the USA [4]. In 2000, an international outbreak of IMD due to MenW occurred during the annual Hajj pilgrimage to Mecca in Saudi Arabia. The outbreak was found to be caused by a MenW strain ST-11 [5, 6]. The Hajj pilgrimage in 2001 resulted in an almost identical outbreak of the same strain [4]. As the Hajj pilgrims returned to their home countries, hypervirulent MenW ST-11 strains likely disseminated throughout the world [7, 8]. However, as of 2002, no further Hajj-related outbreaks were observed after the MenACWY meningococcal vaccine became a requirement for the Hajj visa in Saudi Arabia [8].

In the following years, MenW ST-11 cases remained low at endemic levels through most of the early 2000s. The strain eventually reemerged as two predominant sublineages: the Anglo-French Hajj strain and the South American/UK strain [9]. It is unclear whether these two different strains reflect the emergence of MenW ST-11 in two different geographic regions or ultimately resulted from one common ancestor in 2000 (i.e. the Hajj 2000 strain) [5]. The Hajj outbreak strain was found to be closely related to the Anglo-French Hajj strain but genetically and epidemiologically distinct from the South American/UK strain [9]. Although there have been no reports of Hajj-associated MenW cases in Latin America, surveillance was largely absent in many countries in the years following the Hajj outbreaks. However, the South American/UK MenW ST-11 strain emerged across the Southern Cone of South America during the 2000s. Notable increases in confirmed cases were observed in Argentina, Chile and Brazil [5]. The South American/UK strain can be further characterised into two distinct strains: the ‘original UK strain’, which rapidly emerged in the UK beginning in 2009, and the ‘2013 UK strain’, a variant outbreak strain which emerged in 2013 and is currently expanding outside the UK. Both the original and 2013 strain belong to ST-11 [10]. The Anglo-French Hajj strain appears to have spread from 2003 onward and persists throughout Africa (2003–2013) and France (2012) [11].

In recent times, serogroups C and A (Africa) have been responsible for most epidemics. Disease caused by serogroups B, C and Y have historically been observed at both endemic and epidemic levels (Europe, North America and South America). However, the prevalence of disease caused by MenW has been increasing in Europe, Africa, North America, South America, Asia and the Middle East [5, 11–15]. The percentage of MenW cases in Australia increased from 8% in 2013 to 39% in 2016 [16] and from 3% in 2012 to 19% in 2016 in Canada [7]. In France, the South American/UK strain was the most frequent MenW cc11 isolate found between 2010 and 2016. Of the two South American/UK MenW strains, a significant increase in the ‘2013 UK’ strain was observed in 2016 [11]; this strain has been associated with an atypical clinical disease presentation: some patients have been reported to initially present with acute gastrointestinal (GI) symptoms (i.e., abdominal pain, vomiting and diarrhoea), septic arthritis, severe respiratory infection and peritonitis. The unusual nature of these symptoms has led to delays in IMD diagnosis, with some reports of patients being sent home with a diagnosis of gastroenteritis or undergone appendectomies to address their symptoms [3]. The current review was conducted to further evaluate the reports of MenW IMD cases associated with atypical clinical symptoms on a global scale.

## Methods

### Literature search

A basic PubMed search was performed to identify commonly used terms in literature describing meningitis, serogroup W and atypical presentations. Terms referring to meningococcal disease due to serogroup W were selected in reference to the existing literature, and authors conducted a stepwise literature search with different combinations of these terms to optimise the search string and obtain the maximum number of publications. Terms that did not change the number of publications retrieved were omitted from the final search string. Following this, a literature search was conducted using the Ovid MEDLINE® database (January 1, 1946‒January 23, 2019). The official search terms were created to include all variations or synonyms, and no language limits were applied. The search query was ‘(1 or 2) and 3 and 4’ where 1, 2, 3 and 4 were defined as follows:

1. GI diseases or gastroenteritis
2. Pneumonia or septic arthritis or respiratory or epiglottitis or supraepiglottitis or abdominal or abnormal or unusual or atypical
3. Meningitis or meningococcemia or meningococcal or *N. meningitidis*
4. Serogroup W or group W or MenW or sequence type or ST-11

No filters were applied. Following the search, additional references were added from the authors' personal files if they were relevant to the topic and helpful for discussion.

## Results

### Search results

**Fig. 1.**
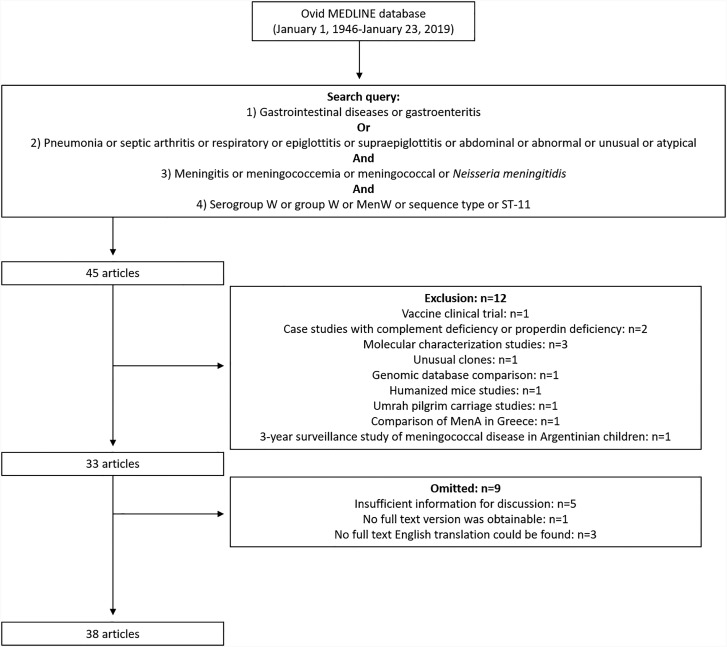
Flow diagram of the study.

The search was performed on January 23, 2019 and yielded 45 results (Fig. 1). The citations were screened by reading their title and abstract. Their relevance to MenW epidemiology, characterisation or clinical presentation provided the basis for inclusion. Twelve articles were excluded for varying reasons; these articles included vaccine clinical trials (1), case studies with complement deficiency or properdin deficiency (2), molecular characterisation studies (3), unusual clones (1), genomic database comparison (1), humanised mice studies (1), Umrah pilgrim carriage studies (1), a comparison of MenA in Greece (1) and a 3-year surveillance study of meningococcal disease in Argentinian children (1). The remaining 33 articles [2–4, 6, 12–15, 17–41] were reviewed, and those that did not provide enough relevant information for discussion or for which an English translation was inaccessible were omitted [17, 19, 23–25, 29, 31, 37, 40]. An additional 14 articles were included from the authors' personal files that were not retrieved by the literature search [1, 5, 7–11, 16, 42–47]. Table 1 provides a summary of the articles retained in this analysis.

**Table 1.** Key messages from articles included in the review

Category	Reference	Key message
MenW IMD studies/reviews	Campbell *et al*. [22]	Among 15 MenW IMD cases in healthy 15‒19-year-olds in England from 2015‒2016, seven cases (five fatal) presented with GI symptoms
	Ladhani *et al*. [26]	In a review of 129 cases of MenW disease in England and Wales (2010‒2013), clinical presentation was often atypical and included septic arthritis and severe respiratory tract infections
	Moreno *et al*. [28]	24% of MenW IMD cases in Chile in 2012 were initially misdiagnosed as gastroenteritis, and disease was fatal in over 50% of these misdiagnosed cases
	Paul *et al*. [14]	In the UK in 2014/2015, MenW IMD was fatal in 12% of cases, which was higher than reported rates for MenB; similar increases have also been observed in other European countries
	Pace *et al*. [1]	Disease presenting as arthritis has been associated with ST-11
	Campbell *et al*. [21]	The proportion of IMD caused by serogroup W increased each year from 2009 to 2015 across all age groups in England, primarily owing to expansion of the ST-11 strain
	Kelly *et al*. [4]	Although MenA had historically been the most common IMD cause in Africa, MenW epidemics throughout the continent became more common following the 2001 Hajj pilgrimage
GI presentations, septic arthritis and pneumonia/respiratory infections	Guiddir *et al*. [3]	Between 2014 and 2016 in France, increased incidence of MenW IMD coincided with increased cases of IMD with abdominal presentation, and the case fatality rate of IMD with abdominal presentation was 24%
	Moll-Manzur *et al*. [27]	In a review of IMD cases in France from 1999‒2002, 26 cases of disease presenting as arthritis were identified, eight of which were caused by MenW; authors found a significant association between arthritis and MenW in contrast to other serogroups
	Vienne *et al*. [43]	A statistically significant association between MenW strains and arthritis was observed, and over half of strains identified in IMD patients presenting with pneumonia belonged to serogroup W
Case reports	Brandstetter *et al*. [20]	Five adult patients with MenW infection presented with clinical manifestations including pneumonia and acute peritonitis
	Cheddani *et al*. [42]	A patient with MenW IMD in France presented with nonspecific GI symptoms as a form of acute, severe sepsis
	Mori *et al*. [13]	A patient with MenW ST-11 infection developed sepsis, disseminated intravascular coagulation, and neurological complications including abducens palsy, cerebellitis and cerebellar infarction
	Brawley *et al*. [41]	A US infant was diagnosed with MenW presenting as septic arthritis
	Iversen *et al*. [15]	A patient with MenW IMD in Denmark presented with abdominal pain diagnosed as bacterial peritonitis and bacteremia
	Seiberras *et al*. [35]	In France in 2005, a 92-year-old patient was diagnosed with MenW disease presenting as pneumonia
	Russcher *et al*. [34]	In 2017 in the Netherlands, a patient was diagnosed with necrotising fasciitis caused by MenW
	Rosas *et al*. [32]	In Chile in 2013, three patients with MenW presented with respiratory infections
	Witt *et al*. [39]	In the United States two patients with MenW were diagnosed with bacteremic meningococcal pneumonia
Molecular	Barra *et al*. [12]	An increased number of IMD cases in Chile in 2010‒2011 were caused by a MenW strain that was related to the Hajj 2000 outbreak strain
	Lucidarme *et al*. [9]	Two sublineages of MenW strain ST-11 were identified, including one containing the South American/UK strain
Epidemiology	Campbell *et al*. [44]	An adolescent MenACWY vaccination program was introduced in the UK in 2015 in response to the increased incidence of MenW disease
	Rubilar *et al*. [33]	A high rate of MenW ST-11 IMD cases in Chile suggested a hypervirulence of this strain compared with other circulating strains
	Tekin *et al*. [36]	In Turkey in 2015, meningococcal carriage among adolescents/young adults peaked at age 17, and serogroup W was the most commonly isolated serogroup
	Parikh *et al*. [30]	Among IMD cases in England from 2011‒2015, atypical clinical presentations were observed for 7.2% of cases; prevalence increased with age from <2.5% in children to 27.6% in older adults, and MenY and MenW accounted for two thirds of atypical presentation cases
	Knol *et al*. [45]	There was an increase in MenW disease incidence in the Netherlands from pre-2015 to 2017, leading to MenACWY vaccination replacing MenC vaccination
	Abad *et al*. [5]	The percentage of MenW cases has increased in the Southern Cone of Latin America, leading to MenACWY vaccination being implemented in Chile
	Knol *et al*. [10]	By 2016 in England and the Netherlands, increased outbreaks were observed caused by the UK 2013 MenW strain; MenW disease was associated with higher case fatality rates (11%) and higher rates of atypical clinical manifestations (25%) compared with MenB disease in the Netherlands
	Hong *et al*. [11]	There was an increase in the spread of the UK 2013 MenW strain in France in 2015‒2016
	Tsang *et al*. [7]	The percentage of IMD caused by MenW increased in Canada between 2012 and 2016, associated with a greater prevalence of strain ST-11
	Wilder-Smith *et al*. [8]	Clusters of MenW IMD cases appeared in Singapore following the Hajj pilgrimage, but the disease was effectively controlled and did not become endemic to the region
	Martin *et al*. [16]	In Australia, the incidence of MenW disease increased from 2015 to 2016, and MenW was responsible for 6 of 8 total IMD deaths that occurred between January 1 and October 5, 2016
	Villena *et al*. [47]	The IMD case fatality rate increased following a MenW outbreak in Chile in 2012, and national adolescent cases of MenW IMD were increasing annually as of 2016
	Batista *et al*. [2]	Atypical presentations of IMD may include primary pneumonia, septic arthritis, chronic meningococcemia, primary pericarditis and others including peritonitis and epiglottitis; septic arthritis and primary pericarditis are primarily associated with serogroups C and W
Miscellaneous	Wang *et al*. [38]	A retrospective analysis of data from 115 patients in Taiwan from 2001‒2003 showed a statistically significant association between MenW and pneumonia
	Apicella *et al*. [18]	A survey of strains isolated in France from 1999‒2001 revealed that pericarditis, arthritis, and pneumonia were more commonly associated with MenW versus MenB or MenC infection

IMD = invasive meningococcal disease; MenACWY = meningococcal serogroups A, C, W-135 and Y; MenB = meningococcal serogroup B; MenC = meningococcal serogroup C; MenW = meningococcal serogroup W; MenY = meningococcal serogroup Y.

As expected, IMD caused by MenW was found to be associated with the presentation of atypical signs and symptoms. The most prevalent atypical symptoms are discussed in the following sections.

### Acute GI symptoms

Although nausea, vomiting and diarrhoea are all symptoms associated with IMD, acute GI symptoms as a primary presentation are rare [22] and considered nonspecific. These nonspecific symptoms may appear during the early stages of meningococcal disease progression but are rarely considered to be the principal signs of IMD. In 2015–2016 in the UK, there were seven MenW IMD cases that presented primarily with GI symptoms; of these, four were reviewed by a clinician and patients were sent home with a presumed diagnosis of gastroenteritis. Of the seven cases, six strains were identified as ST-11 and one strain was nontypeable; five patients died [22]. In 2012, 60 cases of MenW were reported in Chile, comprising 57.7% of all characterised IMD cases. The most common primary clinical symptoms were fever (60.3%), diarrhoea (55.6%), respiratory/cold symptoms (52.5%) and nausea or vomiting (46.7%). Signs of meningeal irritation (Brudzinski sign and nuchal rigidity) appeared only 8.7% of the time. Diarrhoea was observed at a significantly higher rate in patients who died (55.6%) compared with survivors (26.8%). Of the 60 cases, 24% were originally diagnosed as gastroenteritis due to the primary presentation of abdominal pain and 12% of individuals underwent unwarranted surgery. The case fatality rate (CFR) for the MenW cases was 31.7% (19/60) but was 57% among cases initially diagnosed as gastroenteritis (8/14) [28].

**Fig. 2.**
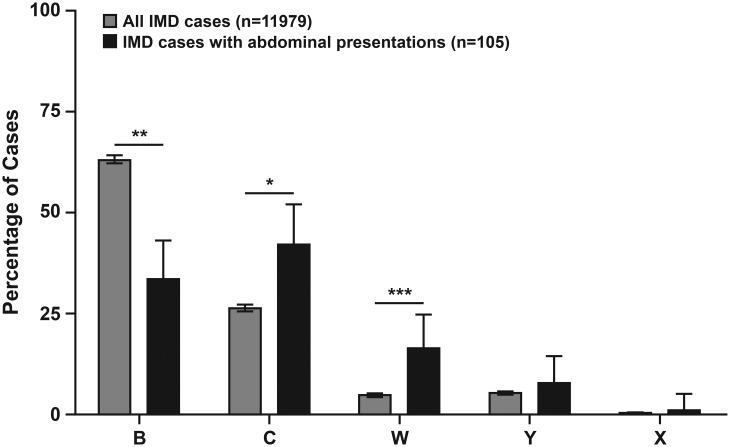
Serotypes responsible for all IMD cases or cases with abdominal presentation in France, 1991–2016. Percentages are provided along with the 95% CIs (*P < 0.01, **P < 0.001, ***P < 0.0001). Adapted with permission from Guiddir *et al*., *Clinical Infectious Diseases* 2018; 67: 1220-1227 [3].

In a third study by Guiddir *et al*. [3], all confirmed IMD cases (*n* = 11 979) in France (1991–2016) were screened for abdominal presentation of IMD based on one or more of the following symptoms within 24 h of being diagnosed: abdominal pain, gastroenteritis with diarrhoea or vomiting or diarrhoea. There were 105/11979 (0.9%) total cases of IMD with abdominal presentation. Of these, MenC, MenB and MenW were responsible for 42%, 33% and 16% of cases, respectively (Fig. 2), and sequencing data were available for 96 strains. ST-11 (MenW and MenC) strains were responsible for 45% of the cases. Further characterisation of 92 of the 105 isolates using MLST showed that all MenW isolates with abdominal symptoms belonged exclusively to the South American/UK strain, suggesting an association between MenW ST-11 and abdominal presentations; this is consistent with the previous two studies demonstrating a significant association between the South American/UK MenW strain and abdominal presentations of IMD in the UK and Chile [22, 28]. During a narrower timeframe of 2010–2016, the proportions of all IMD cases belonging to MenB and MenW (across all STs) *vs.* the proportions of cases exclusively with abdominal presentations belonging to these serogroups were 62% *vs.* 29% for MenB and 6% *vs.* 29% for MenW [3]. Due to the localisation of abdominal pain over the right iliac fossa, unnecessary abdominal surgery was performed in 20% (20/105) of cases in this study [3]. The reported CFR was 24%, compared with a CFR of 10.4% for all IMD cases in France over the same time period [3].

The causal relationship between MenW ST-11 and its presentation in IMD with abdominal pain remains unclear. Changes in genes responsible for metabolic functions in South American/UK and Anglo-French Hajj strains have been demonstrated. Guiddir *et al*. [3] suggest these changes may also affect genes responsible for virulence factors in the meningococcal bacterial wall (lipopolysaccharide and peptidoglycan), which are potent inducers of the inflammatory response; this in turn implies that the induction of an abdominal inflammatory response may be involved in abdominal pain. Previous studies have demonstrated postmortem intestinal inflammation due to MenW IMD [3]. In one case, a 46-year-old man presented to the emergency department with severe acute abdominal pain, vomiting and fever. Before clinicians diagnosed MenW meningococcemia, a computed tomographic scan of the patient's abdomen revealed severe inflammation of the duodenum, and further histologic characterisation showed partial villous atrophy. The authors concluded that abdominal pain experienced by patients with GI symptoms results from mesenteric hypoperfusion and can be improved with the administration of fluids [42].

### Septic arthritis

Reports of meningococcal disease presenting as septic arthritis date back to the 1980s. Brawley *et al*. [41] reported a 22-month-old arriving at the hospital with a swollen, painful knee. Following diagnosis of acute septic arthritis, the infectious agent was identified as MenW. The patient showed no rash or classic signs of meningococcemia, giving the clinician no reason to suspect meningococcal infection [41]. Septic arthritis caused by *N. meningitidis* often presents as pain, erythema, local heat and/or immobilisation or impotence of the joint involved. Septic arthritis tends to be monoarticular, with the knee being the most frequently affected [1], and occurs mainly at the extremes of age [26]. A 3-year clinical follow-up of 129 MenW cases in England and Wales (2010–2013) revealed that septic arthritis was overrepresented in MenW cases, with 9 of 129 IMD cases having MenW strains isolated from joint fluid [26]. A study conducted in France (1999–2002) reported 26 IMD cases with septic arthritis, with MenW ST-11 being responsible for 8 (30.8%) [27].

### Pneumonia and upper respiratory tract infections

Although uncommon, pneumonia caused by *N. meningitidis* can be the primary manifestation of meningococcal disease. Primary meningococcal pneumonia tends to be more common in adults (>50 years old) and associated with serogroups W or Y [1]. Across all serogroups, IMD presenting as primary pneumonia occurs only 5% to 10% of the time [2]. In England and Wales (2010–2015), bacteremic pneumonia was observed in 12% of MenW IMD cases and was more common in individuals ⩾45 years old, at 20%. Surveillance of 3411 IMD cases in England (2010–2015) showed that approximately half (130/235; 55.3%) of the atypical presentations (non-meningitis/septicemia) were pneumonia, with MenW and MenY responsible for two-thirds of the atypical presentations [30]. A study of more than 2000 isolates in France from 1999 to 2002 suggested that MenW is significantly associated with complications of arthritis (*P* < 0.002) and causes pneumonia at much higher rates (18 MenW cases out of 33 total acute pneumonia cases, 54.5%) compared with MenB or MenC [18, 43]. The study also found six cases of meningococcal pericarditis, of which two were caused by MenW, three by MenC and one by MenY [43]. In a Taiwanese study (2001–2003), there was a higher prevalence of pneumonia found in MenW IMD patients *vs.* all IMD patients (23.8% *vs.* 1.5%) [38]. In the United States, two isolated cases of meningococcal pneumonia were described and attributed to MenW [39]. Three cases were described in Chile (2013) in which MenW IMD presented as a primary respiratory tract infection; rapid clinical deterioration with an intense systemic inflammatory response soon followed [32]. All of Chile's MenW IMD cases in 2013 were caused by ST-11 [33].

At early stages, meningococcal disease usually presents as cold-like symptoms, including sore throat, cough, coryza and otalgia [2]. This often results in IMD being indistinguishable from a viral infection in its first 4 to 6 h. Viral infection may make individuals more susceptible to invasion of *N. meningitidis*, leading to IMD. Additionally, damage to the nasopharyngeal epithelium from other factors such as temperature and humidity has been associated with a higher incidence of IMD [1]. MenW IMD has also been associated with a primary presentation of severe upper respiratory tract infection, most notably epiglottitis [22]. In 2010–2013 in England and Wales, five MenW cases presented with a severe upper respiratory tract infection (epiglottitis or supraglottitis), three of which were associated with ST-11 (the remaining two cases were non-ST11 and unknown) [26]. Incidences of severe respiratory tract infections, as well as septic arthritis, were found to be overrepresented among MenW cases. Epiglottitis and supraglottitis are highly rare IMD presentations, and this was the first report of their association with MenW infection [26].

## Discussion

Hajj outbreaks in 2000 and 2001 played a role in disseminating MenW ST-11 across the world, contributing to a rising number of meningococcal disease cases and deaths. MenW continues to evolve as a growing cause of IMD on a global scale. Clinical recognition of meningococcal disease attributed to MenW ST-11 has also been a challenge for physicians because of the higher frequency of unusual clinical presentations compared with typical bacterial meningitis cases. In addition, many of these symptoms are nonspecific. MenW ST-11 was found to be associated with complications such as pericarditis, peritonitis, acute GI symptoms (vomiting, diarrhoea and nausea), septic arthritis and severe respiratory tract infections (pneumonia, epiglottitis) [26]. Although most of these presentations and symptoms are described in all IMD, they have always remained uncommon and rarely seen as the primary presenting sign of IMD.

### What about other ST-11s?

For the Hajj MenW ST-11 outbreak strain (i.e., the current Anglo-French Hajj strain), no link with unusual clinical presentations has been noted. However, cases of MenB and MenC ST-11 have presented with abdominal symptoms, mostly abdominal pain. Of 105 French IMD cases displaying an abdominal symptom (1991–2016), MenC, MenB and MenW were responsible for the majority of cases [3]. The proportion of cases with abdominal presentations compared with all cases was overrepresented for MenC and MenW, but not for MenB (Fig. 2). MenC isolates belonged to several ST-11 lineages, while MenW isolates belonged exclusively to the South American/UK lineage. Thus, these unusual IMD symptoms do not appear to be exclusive to MenW, but may be exclusive to the currently expanding MenW South American/UK ST-11 lineage among MenW strains [3].

### Why the South American/UK strain?

All MenW cases associated with atypical presentations appear to fall within the two sublineages of the South American/UK strain (the original UK strain and the UK 2013 strain); atypical presentations have not been seen in IMD cases caused by the Anglo-French Hajj strain. Whole genome sequencing of the South American/UK and Anglo-French Hajj strains in France (1991–2016) identified differences between the two strains among 119 loci. Of the loci with characterised function, those driving metabolic function (metabolism of carbohydrate, fatty acids, amino acids and nucleic acids) made up the largest proportion. This indicates that several genetic differences may have developed in the emergence of the Anglo-French Hajj and South American/UK strains, potentially resulting in increased transmission and/or expansion. This could explain the recent expansion of the South American/UK strain. Virulence and carbohydrate metabolism have been previously suggested as causative factors of this expansion [3].

### What is causing these atypical presentations?

While several theories including mesenteric hypoperfusion, septic epiploic microinfarctions, immune complex deposition and others have been proposed to explain the pathophysiology behind the observed presentations of abdominal pain, inflammation is suspected to be the more likely culprit. Changes in a strain's virulence factors can induce a stronger inflammatory response. Inflammation of the duodenum has been observed in a previous MenW IMD case with abdominal pain, implying a possible relationship between the two. Polysaccharide W, which makes up the capsule of MenW, has been shown to generate a low immune response, making elimination from the host difficult [38]. This may be a reason why MenW ST-11 strains have been associated with unusual primary infection sites. The factors contributing to the recent emergence of MenW ST-11 characterised by unique clinical symptoms still remain largely unclear and are an area for further investigation.

### Limitations of the included literature

Limited information is available in the literature regarding atypical presentation of MenW disease, and all included studies were either retrospective observational or case reports. In addition, the literature review was subject to selection bias, and although the literature search was designed to maximise the yield of relevant publications, phrasing inconsistencies in the literature likely prevented some relevant articles from being captured. Controlled, prospective studies are needed to inform any unbiased assumptions regarding the differences between serogroup disease presentations.

## Conclusions

This review highlights IMD data from around the world linked with three atypical presentations: acute GI symptoms, septic arthritis and pneumonia. Physicians need to be aware of MenW ST-11's unusual presentations in IMD to allow for recognition and treatment in a timely manner. They should be particularly vigilant with infant and elderly patients, who have a higher incidence of IMD. Clinicians should also be mindful of the possibility when treating any patient in poor condition who resides in an area with a high incidence of MenW IMD. A routine blood or fluid culture may be prudent in any unusual case of gastroenteritis, bacteremic pneumonia or septic arthritis. GI symptoms are an early sign of IMD sepsis and although nonspecific may warrant an observational period in the emergency room. The delay in major differential diagnosis caused by these acute GI symptoms may be a contributor to the high CFR associated with MenW IMD [3] and suggest that atypical primary presentations cause a delay in time to treatment. Moreover, delayed IMD diagnosis or misdiagnosis of IMD cases as viral infections due to atypical primary presentations could consequently delay prescription of antibioprophylaxis and therefore increase risk of secondary cases.

Because the incidence of IMD caused by MenW continues to increase throughout the world, including in Europe, Africa, North America, South America, Asia and the Middle East [5, 11–15], vaccination with MenACWY conjugated vaccines as a protective measure against MenW disease should be considered by national vaccination policies depending on each country's epidemiology and other country-specific factors. Vaccination is also important in the case of MenW disease because of the possibility for missed or delayed diagnosis owing to atypical presentations. Adolescent MenACWY conjugate vaccine programs have been introduced in the UK, Australia and the Netherlands as a result of increasing rates of MenW cases [5, 21, 44–46]. Chile similarly modified its national immunisation program in 2014 to include a single MenACWY dose administered at 12 months of age [47].

In short, the information gathered through this review concludes the following:

- An increase in serogroup W IMD has been observed on a global scale in the past decade, with serogroup W ST-11 strains being largely responsible.
- Serogroup W ST-11 IMD cases have been observed to present with unusual clinical characteristics worldwide.
- These unique presentations include acute GI symptoms, septic arthritis, pneumonia and upper respiratory tract infections.
- As MenW IMD continues to elude rapid diagnosis by physicians due to its frequent atypical presentation, physicians need to be familiar with the atypical signs and symptoms associated with serogroup W meningococcal disease. Quick recognition of this rapidly progressing disease can promote faster treatment and a higher patient survival rate.

## References

[1] Pace D and Pollard AJ (2012) Meningococcal disease: clinical presentation and sequelae. Vaccine 30, B3–B9. 22607896 10.1016/j.vaccine.2011.12.062

[2] Batista RS (2017) Meningococcal disease, a clinical and epidemiological review. Asian Pacific Journal of Tropical Medicine 10, 1019–1029. 29203096 10.1016/j.apjtm.2017.10.004

[3] Guiddir T (2018) Unusual initial abdominal presentations of invasive meningococcal disease. Clinical Infectious Diseases 67, 1220–1227. 29608658 10.1093/cid/ciy257

[4] Kelly D and Pollard AJ (2003) W135 in Africa: origins, problems and perspectives. Travel Medicine and Infectious Disease 1, 19–28. 17291877 10.1016/S1477-8939(03)00019-X

[5] Abad R (2014) Serogroup W meningococcal disease: global spread and current affect on the Southern Cone in Latin America. Epidemiology and Infection 142, 2461–2470. 24831052 10.1017/S0950268814001149 PMC9151320

[6] Centers for Disease Control and Prevention (2001) Update: assessment of risk for meningococcal disease associated with the Hajj 2001. MMWR Morbidity and Mortality Weekly Report 50, 193–199. 11300626

[7] Tsang R (2017) Increase in *Neisseria meningitidis* serogroup W invasive disease in Canada: 2009–2016. Canada Communicable Disease Report 43, 144–149. 29770081 10.14745/ccdr.v43i78a01 PMC5764745

[8] Wilder-Smith A, Chow A and Goh KT (2010) Emergence and disappearance of W135 meningococcal disease. Epidemiology and Infection 138, 976–978. 19845997 10.1017/S095026880999104X

[9] Lucidarme J (2015) Genomic resolution of an aggressive, widespread, diverse and expanding meningococcal serogroup B, C and W lineage. Journal of Infection 71, 544–552. 26226598 10.1016/j.jinf.2015.07.007 PMC4635312

[10] Knol MJ (2017) Temporal associations between national outbreaks of meningococcal serogroup W and C disease in the Netherlands and England: an observational cohort study. Lancet Public Health 2, e473–e482. 29253430 10.1016/S2468-2667(17)30157-3

[11] Hong E (2018) Clonal replacement and expansion among invasive meningococcal isolates of serogroup W in France. Journal of Infection 76, 149–158. 29132919 10.1016/j.jinf.2017.10.015

[12] Barra GN (2013) Molecular characterization of invasive *Neisseria meningitidis* strains isolated in Chile during 2010–2011. PLoS ONE 8, e66006. 23776590 10.1371/journal.pone.0066006 PMC3679051

[13] Mori N (2018) Meningococcal meningitis with neurological complications and meningococcemia due to serogroup W sequence type 11 complex. Journal of Infection and Chemotherapy 24, 398–400. 29373268 10.1016/j.jiac.2017.12.005

[14] Paul SP, Cannon A and Heaton PA (2016) Meningococcal W outbreak. British Journal of Nursing 25, 534. 27231734 10.12968/bjon.2016.25.10.534

[15] Iversen MS (2018) [Spontaneous bacterial peritonitis and bacteraemia caused by meningococci serogroup W clonal complex 11]. Ugeskrift for Laeger 180, V11170841. 29690994

[16] Martin NV (2016) Rise in invasive serogroup W meningococcal disease in Australia 2013–2015. Communicable Diseases Intelligence Quarterly Report 40, E454–E459. 28043219 10.33321/cdi.2016.40.50

[17] Andersen BM and Solberg O (1984) Endotoxin liberation and invasivity of *Neisseria meningitidis*. Scandinavian Journal of Infectious Diseases 16, 247–254. 6436962 10.3109/00365548409070397

[18] Apicella MA (2004) Extrameningeal complications of *Neisseria meningitidis* serogroup W135 infection. Clinical Infectious Diseases 38, 1638–1639. 15156455 10.1086/421030

[19] Bisgaard AK, Fagerberg SK and Hjort U (2017) [Primary septic arthritis is a rare, atypical manifestation of invasive meningococcal disease]. Ugeskrift for Laeger 179, V09170693. 29208200

[20] Brandstetter RD, Blair RJ and Roberts RB (1981) *Neisseria meningitidis* serogroup W 135 disease in adults. Journal of the American Medical Association 246, 2060–2061. 6793741

[21] Campbell H and Ladhani S (2016) The importance of surveillance: group W meningococcal disease outbreak response and control in England. International Health 8, 369–371. 27620924 10.1093/inthealth/ihw037

[22] Campbell H (2016) Presentation with gastrointestinal symptoms and high case fatality associated with group W meningococcal disease (MenW) in teenagers, England, July 2015 to January 2016. Euro Surveillance 21, 30175. 10.2807/1560-7917.ES.2016.21.12.30175 27035055

[23] Harrison LH (2008) Risk factors for meningococcal disease in students in grades 9–12. Pediatric Infectious Disease Journal 27, 193–199. 18277925 10.1097/INF.0b013e31815c1b3a

[24] Joiner KT, Odugbemi TO and Afolabi JK (1990) Disease in children due to serogroup W-135 *Neisseria meningitidis*. African Journal of Medicine and Medical Sciences 19, 1–3. 2109513

[25] Kriz P (2004) Surveillance of invasive meningococcal disease in the Czech Republic. Euro Surveillance 9, 37–39. 15591689

[26] Ladhani SN (2015) Increase in endemic *Neisseria meningitidis* capsular group W sequence type 11 complex associated with severe invasive disease in England and Wales. Clinical Infectious Diseases 60, 578–585. 25389259 10.1093/cid/ciu881

[27] Moll-Manzur C (2016) [Septic arthritis of the knee by *Neisseria meningitidis* serogroup W-135: first case reported in adults]. Medicina Clínica 147, 225–226. 27207241 10.1016/j.medcli.2016.03.025

[28] Moreno G (2013) [Clinical characterization of cases with meningococcal disease by W135 group in Chile, 2012]. Revista Chilena de Infectología 30, 350–360. 24248103 10.4067/S0716-10182013000400002

[29] Olea A (2017) Case-control study of risk factors for meningococcal disease in Chile. Emerging Infectious Diseases 23, 1070–1078. 28628448 10.3201/eid2307.160129 PMC5512488

[30] Parikh SR (2018) Epidemiology, clinical presentation, risk factors, intensive care admission and outcomes of invasive meningococcal disease in England, 2010–2015. Vaccine 36, 3876–3881. 29699791 10.1016/j.vaccine.2018.02.038

[31] Puleston R (2012) An unusual transmission event of *Neisseria meningitidis* serogroup W135 type 2a in a healthcare setting, England, 2012. Euro Surveillance 17, 20308. 23137486

[32] Rosas R (2015) [Respiratory infections and bacteremia caused by *Neisseria meningitidis* serogroup W]. Revista Chilena de Infectología 32, 242–243. 26065462 10.4067/S0716-10182015000300018

[33] Rubilar PS (2018) Increase of *Neisseria meningitidis* W:cc11 invasive disease in Chile has no correlation with carriage in adolescents. PLoS ONE 13, e0193572. 29518095 10.1371/journal.pone.0193572 PMC5843251

[34] Russcher A (2017) Necrotising fasciitis as atypical presentation of infection with emerging *Neisseria meningitidis* serogroup W (MenW) clonal complex 11, the Netherlands, March 2017. Euro Surveillance 22, 30549. 28661395 10.2807/1560-7917.ES.2017.22.23.30549 PMC5479981

[35] Seiberras S and Fourmaux S (2010) [Pneumonia due to *Neisseria meningitidis* W135]. Médecine et Maladies Infectieuses 40, 366–367. 19959310 10.1016/j.medmal.2009.10.007

[36] Tekin RT (2017) The prevalence, serogroup distribution and risk factors of meningococcal carriage in adolescents and young adults in Turkey. Human Vaccines & Immunotherapeutics 13, 1182–1189. 28140784 10.1080/21645515.2016.1268304 PMC5443366

[37] Turhan V (2010) [Meningococcemia and meningitis due to *Neisseria meningitidis* W135 developed in two cases vaccinated with bivalent (A/C) meningococcal vaccine]. Mikrobiyoloji Bulteni 44, 473–478. 21063998

[38] Wang J-L (2006) Clinical features and outcome of sporadic serogroup W135 disease Taiwan. BMC Infectious Diseases 6, 7, doi: 10.1186/1471-2334-6-7. 16420709 PMC1373656

[39] Witt D and Olans RN (1982) Bacteremic W-135 meningococcal pneumonia. American Review of Respiratory Disease 125, 255–257. 6802047 10.1164/arrd.1982.125.2.255

[40] Wunderink HF (2017) [Gastrointestinal symptoms with meningococcal infection. Emergence of *Neisseria meningitidis* serogroup W.]. Nederlands Tijdschr voor Geneeskunde 161, D1456. 28745253

[41] Brawley RL (1980) Acute septic arthritis caused by *Neisseria meningitidis* serogroup W-135. Southern Medical Journal 73, 395–396. 6767281 10.1097/00007611-198003000-00041

[42] Cheddani H (2018) No neck pain: meningococcemia. American Journal of Medicine 131, 37–40. 28821376 10.1016/j.amjmed.2017.08.002

[43] Vienne P (2003) The role of particular strains of *Neisseria meningitidis* in meningococcal arthritis, pericarditis, and pneumonia. Clinical Infectious Diseases 37, 1639–1642. 14689345 10.1086/379719

[44] Campbell H (2015) Targeted vaccination of teenagers following continued rapid endemic expansion of a single meningococcal group W clone (sequence type 11 clonal complex), United Kingdom 2015. Euro Surveillance 20, 21188. 26212140 10.2807/1560-7917.es2015.20.28.21188

[45] Knol MJ (2018) Implementation of MenACWY vaccination because of ongoing increase in serogroup W invasive meningococcal disease, the Netherlands, 2018. Euro Surveillance 23. doi: 10.2807/1560-7917. PMC5915972 29692317

[46] National Centre for Immunisation Research & Surveillance. FactSheet: Meningococcal vaccines. Available at http://ncirs.org.au/sites/default/files/2019-01/Meningococcal%20vaccines%20FAQs_Nov%202018_final.pdf (Accessed 13 December 2019).

[47] Villena R and Santolaya ME (2017) Chilean experience with serogroup W outbreak and meningococcal ACWY conjugate vaccines. *Presented at: 14th Congress of the EMGM, European Meninogococcal and Haemophilus Disease Society. Prague, Czech Republic*.

